# Tamoxifen or aromatase inhibitors with ovarian function suppression in pre-menopausal stage I-III lobular breast cancer

**DOI:** 10.1038/s41523-023-00594-3

**Published:** 2023-10-26

**Authors:** Helena Record, Elle Clelland, Harriet T. Rothschild, Mandeep Kaur, A. Jo Chien, Michelle Melisko, Hope S. Rugo, Firdows Mujir, Laura Huppert, Rita A. Mukhtar

**Affiliations:** 1 Department of Surgery, University of California San Francisco, San Francisco, CA USA; 2 School of Medicine, University of California San Francisco, San Francisco, CA USA; 3 Helen Diller Comprehensive Cancer Center, University of California San Francisco, San Francisco, CA USA; 4 Department of Surgery, Division of Surgical Oncology, University of California San Francisco, San Francisco, CA USA

**Keywords:** Breast cancer, Outcomes research

## Abstract

While adjuvant treatment with the selective-estrogen receptor modulator (SERM) tamoxifen has been the standard of care for pre-menopausal patients with hormone receptor (HR) positive breast cancer, recent trials showed a benefit of aromatase inhibitors (AI) and ovarian function suppression (OFS) for some patients. The approach to endocrine therapy has not been well studied in pre-menopausal patients with invasive lobular carcinoma (ILC). We identified 202 pre-menopausal patients with HR positive stage I-III ILC in an institutional database. We investigated factors associated with endocrine therapy type and determined changes in systemic therapy from 1990–2021. We evaluated associations between endocrine therapy type and disease-free survival (DFS) with a multivariate Cox proportional hazards model. Of 202 patients, most (69.3%) were prescribed a SERM (99.3% tamoxifen). Those who received an AI had significantly higher stage disease. Over time, use of OFS and AI increased significantly in stage II or III cases (from 0% in 1990 to 56% after 2015 for stage II; from 0% to 80% after 2015 for stage III). Concurrently, adjuvant chemotherapy use significantly decreased in stage II cases (from 67% to 19%). In an exploratory multivariable model, longer duration of AI compared to tamoxifen was associated with significantly improved DFS (HR 0.31; 95% CI 0.11–0.86; p = 0.025). While most pre-menopausal patients received adjuvant tamoxifen, the use of OFS and AIs increased significantly over time. The association between AI use and improved DFS may be consistent with prior randomized trials and warrants further investigation into predictive factors to guide treatment selection.

## Introduction

Breast cancer is the most frequently diagnosed cancer in women worldwide^1^. While breast cancer is a heterogeneous disease with different histologic and molecular subtypes, most cases express estrogen receptor (ER) and/or progesterone receptor (PR), making hormone receptor (HR) positive breast cancer the most common subtype overall^2^.

Anti-estrogen endocrine therapies are the backbone of adjuvant systemic treatment for HR positive tumors, with two main classes in use: selective estrogen receptor modulators (SERMs) and aromatase inhibitors (AIs). SERMs such as tamoxifen act as competitive antagonists of ER and can be used in both pre- and post-menopausal patients. In contrast, AIs inhibit peripheral conversion of androgens to estrogen and are ineffective in pre-menopausal people unless combined with suppression of ovarian estrogen production with chemical or surgical ovarian function suppression (OFS).

Consequently, endocrine therapy guidelines for stage I-III HR positive breast cancer vary by menopausal status^3^. For post-menopausal patients, trials show improved survival with the use of AI therapy compared to tamoxifen, making AI the preferred agent in this setting^3,4^. For pre-menopausal patients, tamoxifen was the standard adjuvant endocrine agent until data published in 2014 suggested that incorporation of OFS and AIs showed improvements in disease-free survival (DFS). However, due to quality-of-life considerations and based on a patient’s baseline risk and personal preferences, the choice of adjuvant endocrine therapy in pre-menopausal patients is quite individualized.

Several studies have evaluated the comparative efficacy of different endocrine therapy strategies for pre-menopausal patients with breast cancer^4^. Two large phase III clinical trials (SOFT/TEXT) suggested that OFS in combination with tamoxifen or AI resulted in improved survival outcomes compared to tamoxifen alone^5,6^. This benefit was greatest in high-risk patients (e.g., age <35 years, tumors >2 cm in size, or grade 3)^6^. Current guidelines allow clinicians to choose between OFS/AI, OFS/tamoxifen, or tamoxifen alone based on clinical and molecular features and patient preference, but lack definitive criteria to guide selection^3^.

The choice of optimal adjuvant endocrine therapy may be of particular importance in patients with invasive lobular carcinoma (ILC), the second most common breast cancer histology after invasive ductal carcinoma (IDC). ILC accounts for 10–15% of all breast cancers and differs from IDC in its diffuse growth pattern, resulting from the absence of the adhesion protein E-cadherin^7,8^. ILC is thought to be a particularly hormone driven tumor, with nearly 90% of cases being HR positive^9–11^. Several studies suggest lower efficacy of chemotherapy in ILC, increasing the importance of optimizing endocrine therapy for those with this subtype^12,13^. Data suggest that ER signaling may differ in ILC compared to IDC^14^. Interestingly, while AIs have shown superiority over tamoxifen for patients with breast cancer in general, the relative benefit of AIs over tamoxifen appears to be more pronounced in patients with ILC^15^. Additionally, some pre-clinical data suggest reduced efficacy of tamoxifen in lobular carcinoma^16^.

Given these findings and the paucity of published data on endocrine therapy in pre-menopausal patients with ILC, we characterized practice patterns and impact of type of therapy on outcomes in a single institution cohort of pre-menopausal patients with early-stage ILC. Specifically, we evaluated factors associated with the initial choice of endocrine therapy prescribed, changes in type of endocrine therapy utilized over time, and whether the type of endocrine therapy received was associated with DFS.

## Results

### Study population & baseline characteristics

**Fig. 1:**
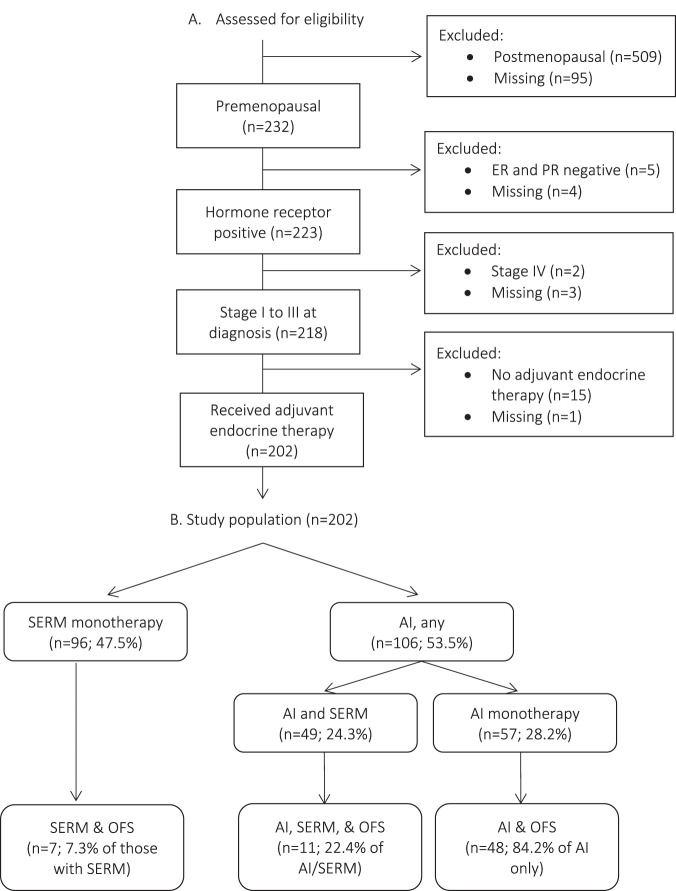
Selection of pre-menopausal patients with HR+ ILC from an institutional database of 835 ILC cases. **A** Flow diagram depicting study population selection from an institutional invasive lobular carcinoma database. **B** Breakdown of type of adjuvant endocrine therapy received throughout treatment. ER estrogen receptor, PR progesterone receptor, SERM selective estrogen receptor modulator, AI aromatase inhibitor, OFS ovarian function suppression.

A total of 202 cases of pre-menopausal patients with early-stage ILC were included in the analysis (Fig. 1). The average age at diagnosis was 47.6 years (range 30–56), and nearly half the cohort (49.5%) had stage II disease (Table 1). Tumor receptor subtype was most commonly ER + PR + HER2- (89.1%), and most tumors were grade 2 (68.6%). Most patients underwent mastectomy (62.9%) and 52.9% of patients received chemotherapy. In total, 114 (56.4%) patients had molecular assay results available. Of those, 86.8% were low risk (RS ≤ 25 or low risk 70-gene assay), and the remaining 13.2% were molecularly high risk. Chemotherapy was used in 14 (93.3%) patients with molecularly high-risk tumors, and 35 (35.4%) patients with molecularly low-risk tumors.

**Table 1:** Patient demographic, tumor, and treatment characteristics by initial endocrine therapy status.

Variable	Total (n = 202)	SERM (n = 140)	AI (n = 62)	p-value
*Demographic & clinicopathologic characteristics*				
Age at diagnosis				
Median age (IQR)	47.6 (6.0)	47.9 (5.9)	46.5 (6.0)	p = 0.64
Histology subtype, n(%)				
ILC	186 (92.1)	126 (90.0)	60 (96.7)	p = 0.100
Mixed ILC/IDC	16 (7.9)	14 (10.0)	2 (3.2)	
Pleomorphic features, n(%)				
Yes	19 (9.4)	13 (9.3)	6 (9.7)	p = 0.930
Lymphovascular invasion, n(%)				
Yes	12 (6.1)	10 (7.5)	2 (3.2)	p = 0.250
Tumor receptor status, n(%)				
ER + PR + HER2-	180 (89.1)	126 (90.0)	54 (87.1)	p = 0.409
ER + PR- HER2-	9 (4.5)	7 (5.0)	2 (3.2)	
ER + PR + HER2+	12 (5.9)	6 (4.3)	6 (9.7)	
ER- PR + HER2-	1 (0.5)	1 (0.7)	0	
Grade, n(%)				
1	51 (26.3)	35 (26.1)	16 (26.7)	p = 0.545
2	133 (68.6)	92 (68.7)	41 (68.3)	
3	10 (5.2)	7 (5.2)	3 (5.0)	
T category, n(%)				
pT1	86 (42.8)	67 (48.2)	19 (30.7)	p < 0.001
pT2	63 (31.3)	48 (34.5)	15 (24.2)	
pT3	52 (25.9)	24 (17.3)	28 (45.2)	
N category, n(%)				
pN0	124 (61.4)	94 (67.1)	30 (48.4)	p = 0.051
pN1	52 (25.7)	31 (22.1)	21 (33.9)	
pN2	15 (7.4)	10 (7.1)	5 (8.1)	
pN3	11 (5.5)	5 (3.6)	6 (9.7)	
Overall stage, n(%)				
I	63 (31.2)	53 (37.9)	10 (16.1)	p = 0.003
II	100 (49.5)	66 (47.1)	34 (54.8)	
III	39 (19.3)	21 (15.0)	18 (29.0)	
*Treatment characteristics*				
Surgical therapy, n(%)				
Lumpectomy + radiation	69 (34.2)	55 (39.3)	14 (22.6)	p = 0.003
Lumpectomy	6 (3.0)	2 (1.4)	4 (6.5)	
Mastectomy	76 (37.6)	56 (40.0)	20 (32.3)	
Mastectomy + radiation	51 (25.3)	27 (19.3)	24 (38.7)	
Chemotherapy, n(%)				
Neoadjuvant chemotherapy	34 (16.8)	21 (15.0)	13 (21.0)	p = 0.296
Adjuvant chemotherapy	75 (37.1)	48 (34.3)	27 (43.6)	p = 0.209
Switched endocrine therapy (SERM & AI)				
Yes	48 (23.8)	45 (32.1)	3 (4.8)	p < 0.001
Adjuvant ovarian suppression, n(%)				
Yes	66 (32.7)	17 (12.1)	49 (79.0)	p < 0.001

*AI* aromatase inhibitor, *ER* estrogen receptor, *PR* progesterone receptor, *SERM* selective estrogen receptor modulator.

P values derived from chi-squared test, t-test, or Wilcoxon rank-sum test as appropriate.

### Factors associated with type of initial endocrine therapy

140 patients (69.3%) were prescribed a SERM as their initial endocrine therapy, whereas 62 patients (30.7%) were prescribed an AI (Table 1). The SERM cohort consisted of patients who received tamoxifen (99.3%), with one patient (0.7%) being prescribed raloxifene. Over the course of treatment, OFS was used in 32% of cases and was significantly more common in those who received an AI than a SERM (79% versus 12.1% respectively, p < 0.001). Of the 12 patients who received an AI without OFS, the majority were noted to become post-menopausal after ILC diagnosis, with 2 (16.7%) experiencing physiologic menopause, 4 (33.3%) experiencing chemotherapy induced menopause, 3 (25%) entering menopause of unclear cause, and 3 (25%) who did not receive OFS for unknown reasons.

Patients who were initially prescribed an AI had significantly larger tumors (median ± interquartile range [IQR]: 4.0 cm ± 2.4 vs. 2.1 cm ± 2.4; p < 0.001), more positive lymph nodes (1 ± 3 vs. 0 ± 1; p = 0.01), and a higher stage of disease (15% stage III vs. 29% stage III, p = 0.003) compared to those who initially received tamoxifen (Table 1). Local and systemic therapy also differed between the two groups. Those in the AI cohort were more likely to undergo mastectomy and radiation, while those in the tamoxifen cohort were more likely to undergo lumpectomy and radiation (p = 0.003).

There was no statistically significant difference in chemotherapy use in those that received tamoxifen versus an AI (46.4% versus 58.1%; p = 0.13). Among the subset of patients who received OFS (n = 66), chemotherapy was utilized in 54.6% (47.1% in the SERM + OFS group, and 57.1% in the AI + OFS group). There was no difference in age, histologic subtype (classic versus mixed ILC/IDC), pleomorphic features, lymphovascular invasion, tumor receptor subtype, or tumor grade between the SERM and AI groups.

### Patterns of systemic therapy use over time

**Fig. 2:**
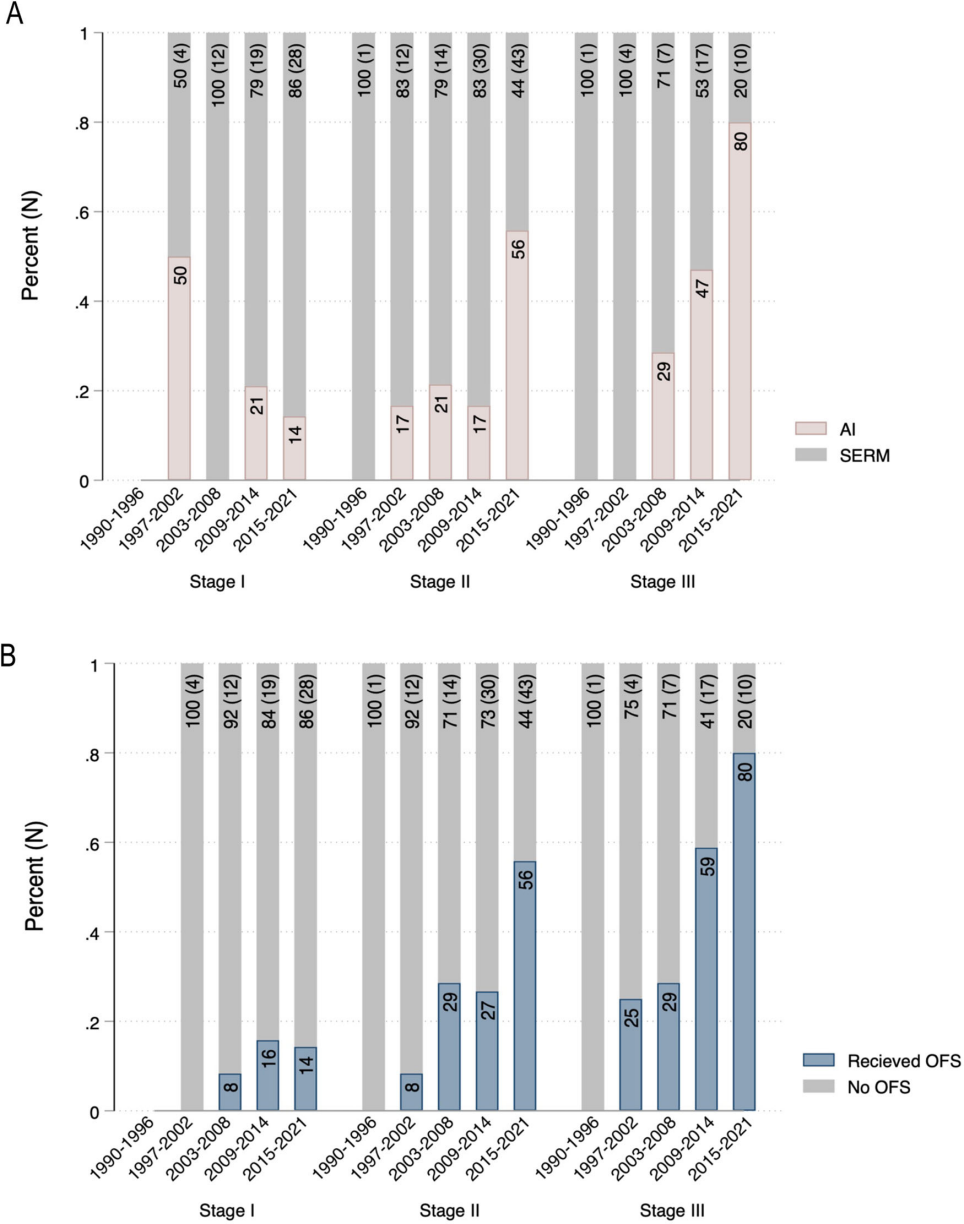
Adjuvant endocrine therapy treatment patterns across year of diagnosis, stratified by cancer stage. **A** Temporal trends in initial type of adjuvant endocrine therapy received (AI vs. SERM). **B** Temporal trends in use of ovarian function suppression. AI, aromatase inhibitor; SERM, selective estrogen receptor modulator; OFS, ovarian function suppression; N, total number of patients diagnosed within time interval.

During the study period, use of an AI as initial adjuvant endocrine therapy increased linearly with year of diagnosis for those with stage II (p = 0.001) and stage III (p = 0.002) disease (Fig. 2A). In stage I cases, there was no difference in the proportion of patients receiving an AI over time. For those with stage II and stage III disease, the largest increase in AI use occurred after 2014. Specifically, among patients with stage II disease, the proportion of patients receiving an AI increased from 17% in the preceding time period to 56% after 2014. Similarly, for those with stage III disease, the proportion of patients receiving AI increased from 47% to 80% after 2014.

Concomitant with an increase in the use of AI, the use of OFS increased over time. The first patient in our cohort who received OFS was diagnosed in 2001. Since then, there has been a significant linear increase in the use of OFS (p < 0.001), especially in those with stage II (p < 0.001) and stage III (p = 0.008) disease (Fig. 2B). After 2014, 56% of patients with stage II disease and 80% of patients with stage III disease received OFS, compared to 27% and 59% respectively for the preceding time period. After adjusting for year of diagnosis, those with higher stages of disease remained significantly more likely to receive OFS: Stage II and III cases had 2.5 and 4.7 times the odds of receiving ovarian suppression, respectively, compared to stage I cases (stage II OR = 2.49, 95% CI 1.2–5.0, p = 0.011; stage III OR = 4.69, 95% CI 1.8–12.0, p = 0.001).

**Fig. 3:**
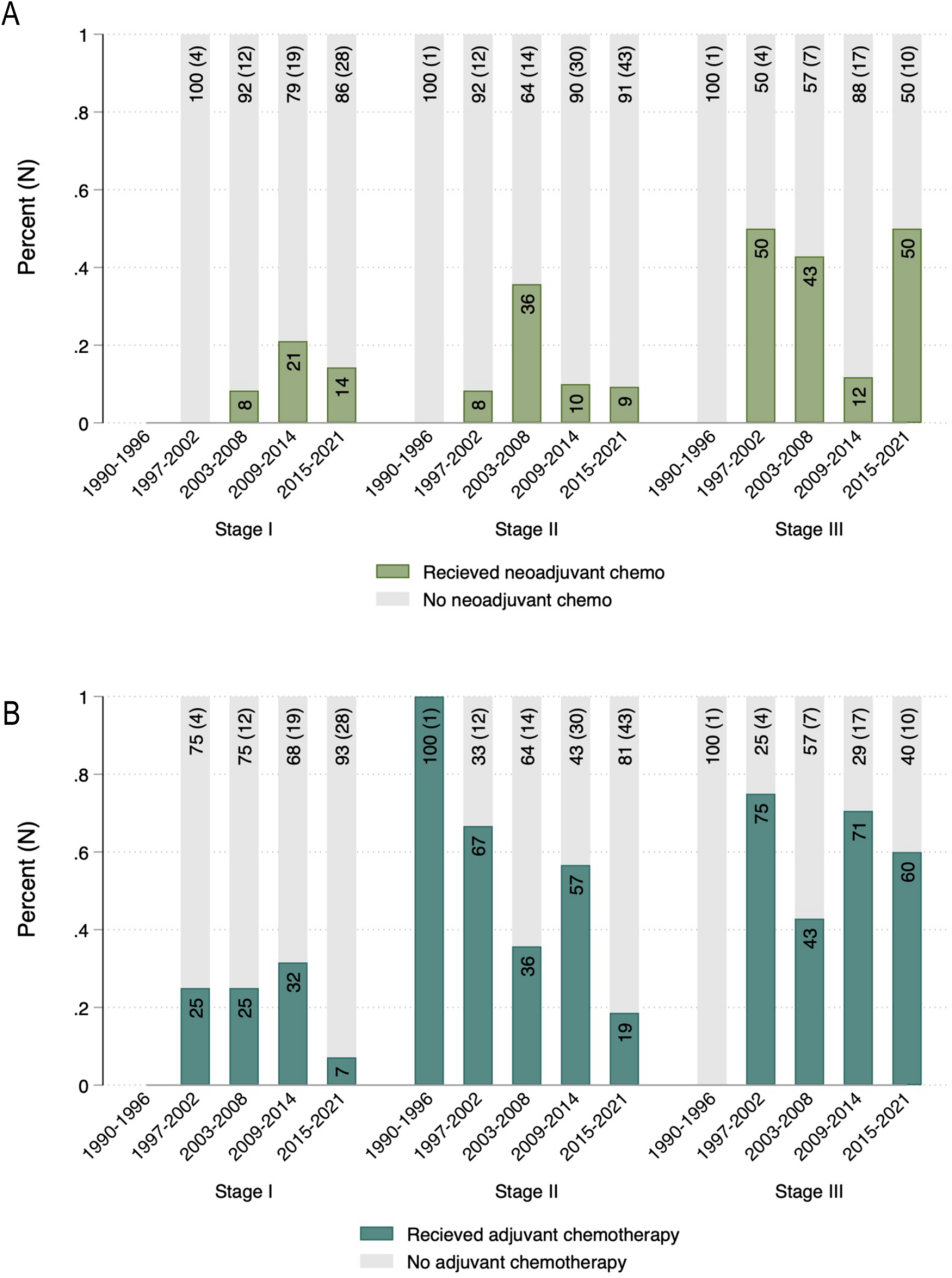
Distribution of chemotherapy use across year of diagnosis, stratified by cancer stage. **A** Temporal trends in neoadjuvant chemotherapy. **B** Temporal trends in adjuvant chemotherapy. N, total number of patients diagnosed within time interval.

We also evaluated temporal trends in the use of neoadjuvant and adjuvant chemotherapy. For patients with stage I or II disease, the use of neoadjuvant chemotherapy remained low over the study period, ranging from 8–36% (Fig. 3A). Among those with stage III disease, neoadjuvant chemotherapy was more common but also did not change over time. In contrast, we found a significant linear decrease in the rate of adjuvant chemotherapy for patients with stage II disease (p = 0.001), but no difference in the use of adjuvant chemotherapy for those with stage I or III disease (Fig. 3B).

### Patients who changed initial endocrine therapy

Among the initial tamoxifen cohort, 45 patients (32.1%) subsequently switched to AI therapy. In this group, 78.4% had longest exposure to tamoxifen, while 21.6% had longest exposure to AIs (and were thus classified in the AI group for survival analyses). Among the initial AI cohort, 3 patients (4.8%) subsequently switched to tamoxifen. In this group, 98.4% had longest exposure to an AI, while 1.6% had longest exposure to tamoxifen (and were thus classified in the SERM group for survival analyses). For patients who had a change in therapy but were classified in the longer duration SERM group, mean duration of treatment with a SERM was 66.2 months, versus a mean of 15.6 months of AI. For those with change in therapy who were classified in the longer duration AI group, mean duration of treatment with an AI was 73.4 months, versus a mean of 25 months of SERM.

### Associations between type of endocrine therapy based on longest duration received and recurrence-free survival

After a median follow up time of 5.9 years (IQR 8.4), 5 deaths occurred, all in the SERM cohort. There were 27 recurrence events, 19 (17.9%) in the SERM cohort and 8 (9.0%) in the AI cohort. Follow-up time did not differ significantly between groups, with a median of 6.2 years (IQR 7.4) in the SERM cohort and 4.9 years (IQR 9.3) in the AI cohort (p non-significant). On univariable analysis, there was no statistically significant difference in Kaplan Meier survival estimates of DFS in the SERM and AI cohorts. However, in a multivariable Cox proportional hazards model adjusting for age at diagnosis, tumor grade, tumor size, number of positive nodes, and receipt of ovarian suppression, those in the AI cohort had a 69% reduction in the risk of recurrence or death relative to the SERM cohort (HR 0.31; 95% CI 0.11–0.86; p = 0.025, Table 2).

**Table 2:** Factors associated with cancer recurrence or death by multivariable Cox proportional hazards model.

Variable	Hazard Ratio	95% CI	p-value
Adjuvant endocrine therapy (longest received)			
SERM			
AI	0.31	0.11–0.86	0.025
Age at diagnosis (above & below median)			
30–46 years old			
47–56 years old	0.61	0.24–1.56	0.304
Histologic grade			
Grade 1 or 2			
Grade 3	6.63	1.4–31.3	0.02
Tumor size			
Tumor size, cm	1.02	0.88–1.19	0.773
Lymph node positivity			
Number of positive nodes	1.10	1.03–1.18	0.003
Receipt of ovarian suppression			
No OFS			
Received OFS	3.77	1.33–10.7	0.013

*AI* aromatase inhibitor, *SERM* selective estrogen receptor modulator, *OFS* ovarian function suppression.

## Discussion

In this study of 202 pre-menopausal patients with stage I-III ILC, we identified changes in practice patterns regarding type of adjuvant therapy used and potential associations between type of endocrine therapy and disease-free survival. Overall, the use of OFS and AIs has increased significantly over time for patients with stage II and III ILC, while the use of adjuvant chemotherapy decreased for those with stage II disease. In a multivariable model, receipt of AI was associated with significantly improved DFS compared to patients on tamoxifen when adjusting for receipt of OFS.

Although ILC is a largely HR positive tumor type known to be associated with prolonged estrogen exposure, there are no published data to our knowledge examining type of endocrine therapy and outcomes for pre-menopausal patients with ILC specifically. While data suggest that the use of OFS + AI or OFS + tamoxifen reduces the risk of recurrence for pre-menopausal patients with breast cancer compared to tamoxifen, this treatment comes at the cost of significant toxicity, with some studies showing poor tolerability of OFS or AI. However, whether the relative benefit of OFS + AI over tamoxifen in pre-menopausal patients differs by histologic subtype may warrant further study^15^.

We found that pre-menopausal patients were more likely to receive an AI if they had larger tumors or more nodal involvement; interestingly, we did not find an association between type of endocrine therapy and younger age, tumor grade, or pleomorphic subtype. The association between higher stage and receipt of AI is consistent with findings from the SOFT/TEXT trials, in which patients with higher risk disease experienced greater benefit from OFS + AI. The dramatic increase in the use of AI for stage II and III disease after the year 2014 coincides with publication of the SOFT/TEXT studies, so we suspect that these trial results contributed to a change in practice at our institution.

Although we did not find an association between receipt of AI and use of chemotherapy in this cohort, we did identify a significant temporal trend in the use of chemotherapy; specifically, the use of chemotherapy in pre-menopausal patients with stage II disease significantly decreased over time. Over the study period, the introduction of genomic assays that predict chemotherapy benefit such as the 21-gene Recurrence Score and the 70-gene assay likely impacted patient selection for such treatment. The majority of ILC tumors are classified as molecularly low risk, with a prior analysis of the National Cancer Database showing that for women under age 50 with HR positive HER2 negative ILC, over 90% had 21-gene Recurrence Scores ≤ 25^17^. While low risk scores may have led to a decrease in the use of chemotherapy for some patients with ILC in our cohort, the recent RxPonder Trial found a significant benefit of chemotherapy in pre-menopausal patients with Recurrence Scores ≤ 25 but 1–3 positive nodes^18^. Given low rates of OFS in the RxPonder study, investigators have suggested that the benefit of chemotherapy in these patients may have been mediated by chemotherapy-induced menopause^19^. As such, the impact of chemotherapy versus AI + OFS in pre-menopausal women with ILC should be explored, particularly for those with stage III disease where both chemotherapy and AI use was common in this cohort. Although not specific to ILC, the NRG Oncology trial NRG-BR009 (OFSET Chemo) plans to prospectively evaluate whether adjuvant chemotherapy improves outcomes in pre-menopausal women receiving OFS plus AI for HR positive HER2 negative breast cancer^20^.

We did note that among the patients in this cohort who initially received AI, 21% did not receive OFS. For the majority, this was explained by the occurrence of physiologic or chemotherapy-induced menopause after diagnosis but before the initiation of adjuvant endocrine therapy. Overall, OFS use increased in stage II and III, mirroring trends in AI use in these subpopulations. However, the absence of OFS in 21% of patients who initially received AI suggests that some patients in this cohort were peri-menopausal and not truly pre-menopausal at diagnosis. This may have impacted the findings regarding improved DFS in the AI group, particularly if peri- or post-menopausal patients have improved outcomes over pre-menopausal patients. It is important to note that there was no difference in age at diagnosis between the tamoxifen and AI groups (47.9 years versus 46.5 years respectively). Additionally, some data suggest that patients with breast cancer and earlier menopause have increased risk of recurrence; this would increase the risk of a DFS event in the AI group, biasing the results of the multivariate model towards the null hypothesis^21^. However, due to the retrospective design and small sample size of this study, this survival analysis was purely exploratory. Results of our adjusted Cox regression model demonstrated that the AI cohort had a 69% reduction in the risk of invasive breast cancer recurrence or death relative to the SERM cohort, potentially consistent with findings from randomized trials^6^. This finding was true even when adjusting for stage and grade and should be validated in larger studies.

Interestingly, receipt of OFS was associated with shorter DFS in the multivariable model; we chose to examine OFS as variable separate from AI, as there were some patients who received tamoxifen plus OFS, but small sample size did not allow us to meaningfully analyze groups defined as tamoxifen with or without OFS and AI with or without OFS. A recent presentation including nearly 15,000 patients from the Early Breast Cancer Trialists’ Collaborative Group demonstrated a significant improvement in recurrence risk for pre-menopausal patients who received ovarian ablation/suppression, with a reduction in the absolute magnitude of benefit in those who received tamoxifen versus no tamoxifen^22^. We suspect that the association between OFS and shorter DFS in our cohort reflects the retrospective nature of this study, where patients with higher risk disease were selected to receive OFS.

Strengths of this study include access to a prospectively maintained institutional ILC database that contains treatment and outcomes data for over 200 pre-menopausal patients with ILC diagnosed over the past thirty years. This dataset afforded us the unique opportunity to evaluate changes in practice patterns over time and allowed for menopause status to be defined by physician documentation rather than estimated by patient age, although diagnostic criteria for pre-menopausal status were not pre-defined. Additionally, the institutional nature of this study has inherent weaknesses, including limited generalizability outside of large academic centers in the United States. It was also not possible to compare trends in endocrine therapy use and outcomes between ILC and IDC using these data, so future work is needed to better understand differences between these groups.

In summary, while we found that the majority of pre-menopausal patients with ILC in this study initially received tamoxifen as their adjuvant endocrine therapy, the use of both AI and OFS has increased significantly in recent years, especially after 2014, for those with stage II and III disease. Concurrent with this is a significant decrease in chemotherapy use for those with stage II disease. These findings highlight the need for ILC specific studies to help determine the optimal endocrine therapy approach, and predictors that may help identify patients likely to benefit the most from OFS/AI. Our findings reflect real-world application of the SOFT/TEXT trial data, applied to a population with ILC. The finding of improved DFS in pre-menopausal patients with ILC who received an AI is reassuring, and further investigation into the impact of chemotherapy on pre-menopausal patients with early stage ILC may help further refine patient management.

## Methods

### Study design & data source

In this retrospective cohort study, we queried a prospectively maintained institutional database containing treatment and outcomes data for 835 patients with ILC who underwent surgical treatment at the University of California, San Francisco (UCSF) between January 1990 and October 2021. Data were gathered from electronic medical records by manual extraction and analysis was approved by the UCSF Institutional Review Board. A waiver of informed consent was granted given the retrospective nature of the study. Authors complied with all relevant ethical regulations including the Declaration of Helsinki.

### Study population

We included all cases of pre-menopausal patients diagnosed with HR positive, stage I-III ILC or mixed ILC/IDC who received adjuvant endocrine therapy (Fig. 1). Pre-menopausal status was defined at the time of ILC diagnosis and determined from medical oncology or primary care notes, based on date of last menses or estradiol levels if available. Hormone receptor positivity was defined as either estrogen or progesterone receptor positivity, with ≥1% staining on immunohistochemistry considered positive. Type and duration of adjuvant endocrine therapy was assessed by review of the medical record.

### Study variables, outcomes, and covariates

For analysis of temporal trends in type of systemic therapy used, cases were categorized by year of diagnosis. The study period of 1990 to 2021 was analyzed in intervals of 6–7 years to allow comparison before and after publication of the SOFT/TEXT trial results in 2014 and early 2015 (i.e, 1990–1996, 1997–2002, 2003–2008, 2009–2014, and 2015–2021). Patients were then grouped in two ways: first, by the initial type of endocrine therapy received (AI or SERM), and second, by the type of endocrine therapy received for the longest duration (to account for patients who switched from one type of endocrine therapy to another). When available, we recorded molecular assay results (21-gene Recurrence Score or 70-gene assay); tumors with either a 21-gene Recurrence Score (RS) of ≤25 or a 70-gene assay reported as “low risk” were considered to be low risk.

For analysis of factors associated with receiving AI versus SERM and temporal trends in the proportion of patients receiving AI versus SERM, we utilized groups based on initial endocrine therapy received. We also examined trends over time in the use of OFS and chemotherapy, stratified by pathologic stage.

As a secondary aim, we examined DFS by type of endocrine therapy received for the longest duration. DFS was defined as the time from ILC diagnosis to the first occurrence of invasive breast cancer recurrence (local or distant) or death from any cause; patients without recurrence events or death were censored at the date of last follow up. To account for confounding, a graphical causal model (i.e., directed acyclic graph or DAG) was constructed based on existing literature and clinical knowledge^23^.

### Statistical analysis

Differences in patient demographic, tumor, and treatment characteristics were compared by type of initial endocrine therapy received using a chi-squared test, t-test, or Wilcoxon rank-sum test, as appropriate. Changes in the use of systemic therapies (adjuvant endocrine therapy, OFS, chemotherapy) across the study period were evaluated using bar graphs, logistic regression, and Cochrane Armitage trend tests, as appropriate.

Unadjusted time-to-event outcomes were estimated and plotted using the Kaplan-Meier method, and survival curves were compared using a log rank test. Disease-free survival was analyzed using a multivariable Cox proportional hazards model and adjusted for confounding variables identified by causal modeling. All time-to-event analyses met proportional hazards assumption as assessed by the Schoenfeld residuals. Missing information was considered missing at random. All tests were 2-sided, and p-values of $\le 0$.05 were considered statistically significant. Analyses were performed using Stata 17.

### Reporting summary

Further information on research design is available in the Nature Research Reporting Summary linked to this article.

### Supplementary information

Reporting Summary

## Data Availability

The data supporting all tables and figures in this published article are not publicly available to protect patient privacy but can be accessed from the corresponding author on request. Data will be made available to authorized researchers who have obtained Institutional Review Board (IRB) approval from their own institution and from the University of California, San Francisco IRB.

## Code availability

Analysis codes are available from the corresponding author upon reasonable request.
